# Knockdown of *MLO* genes reduces susceptibility to powdery mildew in grapevine

**DOI:** 10.1038/hortres.2016.16

**Published:** 2016-04-20

**Authors:** Stefano Pessina, Luisa Lenzi, Michele Perazzolli, Manuela Campa, Lorenza Dalla Costa, Simona Urso, Giampiero Valè, Francesco Salamini, Riccardo Velasco, Mickael Malnoy

**Affiliations:** 1 Research and Innovation Centre, Fondazione Edmund Mach, via Edmund Mach 1, 38010 San Michele all’Adige, Italy; 2 Wageningen UR Plant Breeding, Wageningen University and Research Centre, PO Box 386, 6700 AJ Wageningen, The Netherlands; 3 Department of Ecological and Biological Sciences, Università della Tuscia, Via San Camillo de Lellis, Viterbo 01100, Italy; 4 Council for Agricultural Research and Economics (CREA), Rice Research Unit, S.S. 11 per Torino km 25, Vercelli 13100, Italy; 5 Council for Agricultural Research and Economics (CREA), Genomics Research Centre, Via San Protaso, 302, 29017, Fiorenzuola d’Arda, Piacenza, Italy

## Abstract

*Erysiphe necator* is the causal agent of powdery mildew (PM), one of the most destructive diseases of grapevine. PM is controlled by sulfur-based and synthetic fungicides, which every year are dispersed into the environment. This is why PM-resistant varieties should become a priority for sustainable grapevine and wine production. PM resistance can be achieved in other crops by knocking out susceptibility S-genes, such as those residing at genetic loci known as *MLO* (*Mildew Locus O*). All *MLO* S-genes of dicots belong to the phylogenetic clade V, including grapevine genes *VvMLO7*, *11* and *13*, which are upregulated during PM infection, and *VvMLO6,* which is not upregulated. Before adopting a gene-editing approach to knockout candidate S-genes, the evidence that loss of function of *MLO* genes can reduce PM susceptibility is necessary. This paper reports the knockdown through RNA interference of *VvMLO6*, *7*, *11* and *13*. The knockdown of *VvMLO6*, *11* and *13* did not decrease PM severity, whereas the knockdown of *VvMLO7* in combination with *VvMLO6* and *VvMLO11* reduced PM severity up to 77%. The knockdown of *VvMLO7* and *VvMLO6* seemed to be important for PM resistance, whereas a role for *VvMLO11* does not seem likely. Cell wall appositions (papillae) were present in both resistant and susceptible lines in response to PM attack. Thirteen genes involved in defense were less upregulated in infected *mlo* plants, highlighting the early *mlo*-dependent disruption of PM invasion.

## Introduction

Vineyards are treated with an impressive amount of chemical compounds, particularly fungicides, to prevent yield losses due to fungal pathogens. In France, Italy, Spain and Germany, between 1992 and 2003, 73% of the fungicides were used for grapevine protection, a crop that covers only 8% of the agricultural land in those countries.^1^

Worldwide, grapevine powdery mildew (PM), caused by the fungus *Erysiphe necator*, is a destructive disease.^2^ *E. necator* Schw. (syn. *Uncinula necator* (Schw.) Burr.) is an obligate biotroph infecting all green tissues of grapevine and results in significant losses in yield and berry quality. Symptoms are a white or gray powder covering both leaf surfaces, and, after infection, the fruits show shriveling or cracking.^3^ The quality of the fruit is severely damaged, due to increased acidity and decreased anthocyanin and sugar content.^4^

PM can be controlled by frequent applications of fungicides, particularly those based on sulfur. However, due to the ecological drawbacks of fungicides,^5^ the relative high costs (up to 20% of total grapevine production expenses^6^) and the rapid appearance of resistant strains of the pathogen^7–9^ because of its adaptative gene copy-number variation,^10^ new alternatives to chemical treatments should be adopted. Resistant varieties are one of the best options. The use of PM-resistant cultivars could reduce production costs in California by 720 $ ha^−1^, with a significant reduction of fungicide usage.^6^

*Vitis vinifera* is susceptible to PM,^11^ whereas North American *Vitis* species, due to their co-evolution with *E. necator,* have variable degrees of resistance to the pathogen.^12^ Their resistances have been transferred to *V. vinifera* but the acceptance of resistant hybrids by producers and consumers has been very limited because of the attachment to traditions and lower quality of resulting wine, although resistant cultivar suitable for wine production are becoming available.^6^ One strategy to create crops resistant to diseases is based on the exploitation of R-genes that encode proteins that recognize pathogen effectors and trigger defense response,^13^ such as the *Vitis REN* and *RUN* genes.^14^ Resistance manifests as localized hypersensitive responses at the sites of attempted infection.^15^ However, R-genes are frequently overcome by mutations of the pathogen.^16^ An alternative approach is based on susceptibility genes (S-genes), which loss of function results in recessively inherited resistance.^17^ Knockout of S-genes may, however, induce pleiotropic phenotypes in the plant.^18,19^

A typical class of S-genes is represented by the *MLO* (*Mildew Locus O*) genetic factors that, when inactivated, results in recessive *mlo* resistance, as discovered in barley.^20^ *MLO* genes are largely conserved across the plant kingdom and their loss of function resulted in PM resistance in *Arabidopsis thaliana*,^21^ pea,^18^ tomato,^22^ wheat^23^ and pepper.^24^ Of the seven phylogenetic clades in which the *MLO* family is divided,^25,26^ only two include S-genes: clade IV with all monocot S-genes^27,28^ and clade V with all dicot S-genes.^21,22,29,30^ Not all members of clades IV and V are S-genes, but candidates can be identified during early stages of PM infection because of their increased expression, as documented in tomato,^22^ barley,^31^ pepper,^24^ grapevine^29,30^ and apple.^26^ In grapevine, of four clade V *MLO* genes, three (*VvMLO7*, *VvMLO11* and *VvMLO13*) are upregulated early after PM infection, whereas *VvMLO6*, the fourth, is not transcriptionally responsive to the pathogen.^29,30^

MLOs are membrane proteins with seven transmembrane domains^32^ involved also in a variety of physiological processes in different tissues, such as root morphogenesis^33^ and pollen tube reception by embryo sac.^34^ The proposed function of *MLO* S-genes is the negative regulation of vesicle-associated and actin-dependent defense pathways at the site of PM penetration.^27^ The secretory vesicle traffic controls pathogen penetration, allowing the formation of cell wall appositions called papillae,^2,33^ which are associated with *mlo* resistance.^21,36^

The development of DNA-editing tools is rapidly changing plant genetics and biotechnology, due to the possibility of inducing mutations in specific genes.^37–39^ Before adopting a gene-editing approach to knockout candidate S-genes, the evidence that loss of function of *MLO* genes can reduce PM susceptibility is necessary. This paper reports the knockdown through RNA interference of *VvMLO6*, *7*, *11* and *13* and its effect on PM infection in grapevine.

## Materials and Methods

### Constructs

Three hundred- to 600-bp fragments of genes *VvMLO6*, *VvMLO7*, *VvMLO11* and *VvMLO13* were amplified (Supplementary Table S1) and cloned in pENTR/SD-TOPO (Invitrogen). After checking the sequences, the fragments were inserted in the RNAi Gateway vector pK7GWIWG2D(II)^40^ (http://www.psb.ugent.be/), as in Urso *et al*.^41^ After sequencing both the strands, the constructs were inserted in *Agrobacterium tumefaciens* strain GV3101, as in Zottini *et al*.^42^ *A. tumefaciens*-transformed cells were tested by PCR for the presence of constructs, using primers annealing to the 35S promoter (5′-CGCACAATCCCACTATCCTT-3′) and the *MLO* fragment (Supplementary Table S1).

### Plant material, gene transfer and screening of regenerants

Plant material of the grapevine cultivar long-cluster Brachetto was cultivated *in vitro* as described by Dalla Costa *et al*.^43^ This cultivar was chosen because it is easy to transform,^44,45^ has high somatic embryogenesis efficiency and good efficiency of regeneration from the callus.^46^ Somatic embryos were used for gene transfer. Gene transfer, regeneration and selection of transgenic plants were performed as in Dalla Costa *et al*.^43^ Five different gene transfers were carried out: four aimed to silence the four *MLO* target genes and the fifth was a control consisting of the empty vector (pK2WG7). DNA was extracted from *in vitro* leaf tissue (Phytopure kit, GE Healthcare, Little Chalfont, Buckinghamshire, UK). Integration was proven using the primers described above. Transformed *in vitro* grown lines were moved to a woody plant medium^47^ in growth chamber at 20–24 °C and transferred in fresh medium once a month.

### Greenhouse acclimation

Plants were first acclimated to greenhouse conditions in a growth chamber at 25 °C, 16-h day/8-h night, relative humidity (RH) 70±5%. One-month-old plants with at least two main roots 3-cm long were transferred in a 250-ml plastic cup containing wet autoclaved turf (Tercomposti Spa, Brescia, Italy) and sealed with parafilm, to preserve humidity. Every 7 days, holes were made in the parafilm cover to progressively reduce air humidity and promote the formation of the foliar cuticle. After 3 weeks, parafilm was completely removed and, after 1 more week, the plants were transplanted in 1 l pots kept in the greenhouse at 25 °C, 16-h day/8-h night, humidity 70±5%.

### *E. necator* and *P. viticola* inoculation and disease severity assessment

The *E. necator* and *P. viticola* inocula were obtained from infected leaves of an untreated vineyard in northern Italy (Trentino region). Subsequent reproduction of the inoculum was carried out infecting the *V. vinifera* cultivar Pinot Noir, under greenhouse conditions. For *E. necator* inoculation, plants were dry inoculated onto target leaves gently brushing adaxial leaf surfaces with infected young leaves carrying fresh PM sporulation.^48^ Inoculated plants were incubated in a greenhouse at 25±1 °C with 100% RH for 6 h to promote fungal penetration, and then kept at 25±1 °C and 70±10% RH until the end of symptom evaluation. Disease severity was assessed on all leaves at 14, 22 and 30 days post inoculation (d.p.i.).

For *P. viticola* inoculation, fresh sporangia were collected by washing the abaxial surfaces, carrying freshly downy mildew (DM) sporulating lesions, with distilled water at 4 °C. Abaxial leaf surfaces were sprayed with the inoculum suspension of *P. viticola* (2×10^5^ sporangia per ml). Inoculated plants were incubated overnight in the dark at 25 °C with 99–100% RH, and then maintained under controlled greenhouse conditions at 25±1 °C and 70±10% RH. Six days after inoculation, plants were incubated overnight in darkness at 25 °C with 99–100% RH to allow DM sporulation and assess the disease severity.

Severity of PM and DM was assessed visually on all leaves of each plant, according to the standard guidelines of the European and Mediterranean Plant Protection Organization.^49,50^ For each leaf, disease severity was expressed as the proportion (percentage of 0–100%, with intervals of 5%) of the leaf area covered by white sporulation of PM or DM in relation to the total leaf area, and a mean value was calculated for each plant. Disease reduction was calculated as (disease severity in control plants−disease severity in transformed plants)/(disease severity in control plants) and expressed as a percentage. For PM severity, the area under disease progress curve was considered as a quantitative summary of disease intensity over time to analyze all time points together^51,52^ using the following formula: area under disease progress curve=∑[(*X_i_* +*X*_*i*+1_)/2]×(*t*_*i*+1_–*t_i_*), where *X_i_* corresponds to the disease severity (%) at assessment *i*, *X*_*i*+1_ corresponds to the disease severity (%) at subsequent assessment *i*+1 and (*t*_*i*+1_−t_*i*_) corresponds to the number of days between the two consecutive assessments. PM severity was also assessed as the number of *E. necator* conidia produced from infected leaves as in Angeli *et al*.^53^ Three leaves were collected from each replicate at 30 d.p.i. and four disks of 0.8 cm diameter for each leaf were cut, for a total of 12 disks per replicate. Leaf disks were transferred to 50-ml tubes containing 5 ml distilled water with 0.01% Tween-80 (Sigma-Aldrich, St. Louis, MO, USA). Tubes were vortexed for 1 min and the concentration of conidia per ml was determined by a hemocytometer count. The values obtained were converted in conidia per cm^2^ of grapevine leaf. Two inoculation experiments were carried out and in each experiment three to nine biological replicates (plants) per line were analyzed in a randomized complete block design.

### Histological analysis

Two inoculated leaves were collected from three replicates of each transgenic and control line at 3, 10 and 21 d.p.i. for hyphae visualization and histological analyses. Leaves were treated as described by Vanacker *et al.*^54^ with the following modifications: small pieces of leaf with the adaxial surface up were laid on filter paper moistened with ethanol:glacial acetic acid (3:1, v/v) until the chlorophyll was removed. Leaf segments were transferred to water soaked filter paper for 2 h, incubated in lactoglycerol [lactic acid:glycerol:water 1:1:1 (v/v/v)] for 12 h and stored in lactoglycerol at room temperature. For microscopic analysis, leaf segments were mounted on microscope slides and a drop of aniline blue (0.1% (w/v) in lactoglycerol) was pipetted on their surface. Aniline blue staining does not fluoresce when in lactoglycerol and *E. necator* structures (hyphae, conidia and appressoria) were visualized using the bright-field illumination of a Leica LMD7000 microscope (Leica Microsystems, Wetzlar, Germany). After spore localization, fluorescence was used with a LMD filter (BP filter 380–420-nm excitation, 415 dichroic mirror, and BP 445–485-nm emission) to visualize the bright blue–green auto-fluorescence associated with infected cells and papillae (autofluorogenic phenolic compounds) formation.^54^

### RNA extraction and gene expression analysis

The first gene expression analysis of transgenic plants was carried out on *in vitro* grown lines to identify genotypes with reduced expression of target genes. Three biological replicates were collected from each line. The second analysis was carried out on acclimated transgenic plants, with leaf samples collected before inoculation, 24 h and 10 days post PM inoculation, the time of the last two samplings corresponding to the upregulation of *MLO* genes after infection.^29,30^ Five biological replicates were collected from each line. For each line at each time point, the third and fifth half leaves from the top were collected, frozen in liquid nitrogen and stored at −80 °C.

Total RNA was extracted with the Spectrum Plant Total RNA kit (Sigma-Aldrich). Following a treatment with the DNAse I (Sigma-Aldrich), the RNA was reverse-transcribed using the SuperScript III reverse transcriptase (Invitrogen, Life Technologies, Waltham, USA).

Quantitative PCR amplification (SYBR Green Supermix, Bio-Rad, Hercules, CA, USA) was carried out in a 15-μl volume (primers in Supplementary Table S2) and the results were recorded by a CFX96 Touch Real-Time PCR detection system (Bio-Rad), run by CFX Manager software (Bio-Rad Laboratories, Hercules, CA, USA). The software applies comparative quantification with an adaptive baseline. Each sample was run in two technical replicates with the following parameters: 95 °C 3 min, 40 cycles of 95 °C 10 s and 55 °C 30 s, with a final step at 95 °C 10 s. Primers for genes *VvMLO6*, *VvMLO11* and *VvMLO13* were taken from Winterhagen *et al.*,^30^ whereas for *VvMLO7* they were specifically designed (Supplementary Table S2). Primers for *VvWRKY19*, *VvWRKY27*, *VvWRKY48* and *VvWRKY52* were taken from Guo *et al.*,^55^ for *VvEDS1* from Gao *et al.*^56^ and for *VvPR1*, *VvPR6* and *VvLOX9* from Dufour *et al.*^57^ The new primer pairs were designed with the NCBI Primer Designing Tool (http://www.ncbi.nlm.nih.gov/tools/primer-blast/; Supplementary Table S2). Complementary DNA samples diluted 10, 100, 1000 and 10 000 times were used to test calculate the efficiency of the primers pairs and the size of the PCR products was confirmed by agarose gel electrophoresis. Presence of a specific final dissociation curve was determined after every quantitative PCR run with progressive increments of temperature from 65 to 95 °C (0.5 °C each step, 5 s).

Reference genes were, as reported for grapevine,^58^ *Elongation Factor 1α*, *GAPDH* and *Actin*. Reference genes stability was assessed with GeNorm (medgen.ugent.be/~jvdesomp/genorm/): the three genes had M-values <0.5, well below the threshold of 1.5 considered sufficient for stability.^59–61^

Threshold cycles (Ct) were converted to relative expression following Hellemans *et al.*^62^ and based on the average Ct of two technical replicates. For *MLO* genes, the reference Ct was the average of all samples; for other genes, the control EVB (empty vector Brachetto) at *T*=0 was adopted.

### Statistical analyses

#### Disease severity

Data were analyzed using the Statistica 9 software (StatSoft, Tulsa, OK, USA) and the package SPSS (IBM, Armonk, NY, USA). The smallest statistical unit considered was a plant. Severity values of all leaves were averaged, resulting in the value considered in further analyses. Normal distributions (Kolmogorov–Smirnov and Shapiro–Wilk tests, *P*>0.05) were validated for variances homogeneity (Levene’s test, *P*>0.05) and subsequently used for one-way analysis of variance (ANOVA) with Tukey’s *post hoc* test (*P*<0.05) at each time point. Data were transformed in arcsin(*x*) to meet the pre-requisites of ANOVA. In case of non-homogeneous variances, the Games–Howell’s *post hoc* test was used.

In some cases, data from two experiments were pooled and the ANOVA applied independently for each time point (14, 22 and 30 d.p.i.). Area under disease progress curve data were treated as for severity data. Conidia counts were analyzed with the Kruskall–Wallis test (*P*<0.05).

#### Quantitative PCR data analysis

Values of relative expression were expressed in logarithms^26^ to obtain normal distributions and homogeneity of variances of the residues, as assessed with Shapiro–Wilk (*P*≤0.05) and Levene (*P*≤0.05). Homoscedastic data were analyzed with Tukey’s test (*P*<0.05) and non-homoscedastic with Games–Howell test (*P*<0.05) using the statistical package SPSS (IBM).

Expression data from two experiments were analyzed independently and pooled. Differences were revealed by one-way ANOVA with Tukey *post hoc* test (*P*<0.05). In addition, a two-way ANOVA with Tukey *post hoc* test (*P*<0.05), considered at the same time the effects of the transgenic line and of the time point. For the gene expression characterization of TLB4, Fisher *post hoc* test was used.

## Results

### Gene transfer, selection and acclimation of *MLO* transgenic lines

A total of five gene transfers were carried out. Four aimed to knockdown (KD) specific *MLO* genes (i=KD-*VvMLO6,* ii=KD-*VvMLO7,* iii=KD-*VvMLO11,* iv=KD-*VvMLO13*), the fifth to insert an empty vector. Thirty-seven regenerated lines were obtained, with 29 of them confirmed to contain the insert (Supplementary Table S3). The result of the PCR analysis of six lines is shown in Supplementary Figure S1. Twenty-six transgenic lines were propagated *in vitro* and tested for the silencing of *MLO* genes with quantitative PCR. Due to *in vitro* contaminations, three lines were lost before it was possible to test the level of expression. Gene knockdown was evident for three lines out of eight from gene transfer (iii) (KD-*VvMLO11*) and three out of nine from gene transfer (iv) (KD-*VvMLO13*), whereas the lines regenerated from gene transfers (i) (KD-*VvMLO6*) and (ii) (KD-*VvMLO7*) did not show any reduction of expression (data not shown). Regenerated lines were also tested for off-target silencing, showing that the RNAi fragments targeted other clade V *MLO* genes (data not shown). Six lines with various combinations of silenced genes were selected and indicated with acronyms TLB1 (transgenic line of Brachetto) to TLB6 (Supplementary Table S3). Lines from TLB1 to 3 came from gene transfer (iii) (KD-*VvMLO11*), lines from TLB4 to TLB6 from gene transfer (iv) (KD-*VvMLO13*; Supplementary Table S3). The control was the EVB line. In addition, TLB7, a regenerated line with no reduction of expression, was also included. All lines, including the control, will be referred in the text as ‘transgenic lines’. Lines from TLB1 to 7 are further indicated as ‘RNAi lines’ and from TLB1 to 6 ‘*mlo* lines’.

The survival rate of plants to the acclimation process was ~85%. Under greenhouse conditions, the transgenic plants showed normal growth and no pleiotropic phenotypes.

### PM and downy mildew resistance of transgenic lines

**Figure 1:**
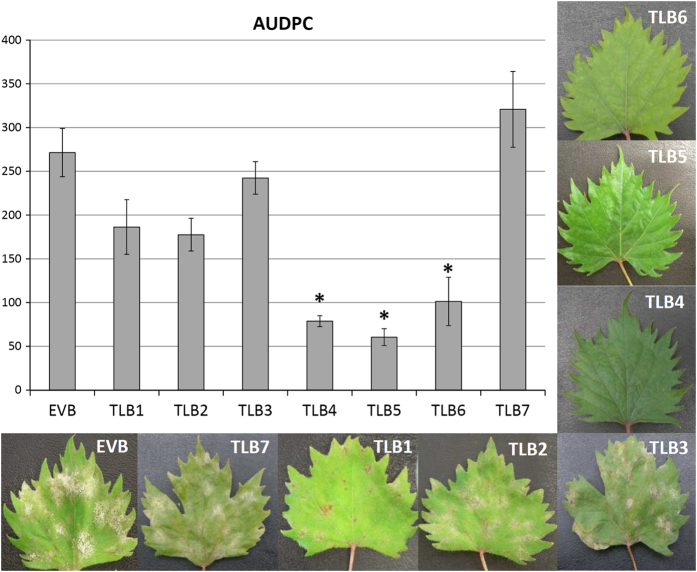
Area under disease progress curve (AUDPC) of grapevines inoculated with *Erysiphe necator* in control (EVB) and transgenic lines (TLB1, 2, 3, 4, 5, 6 and 7). The average scores of AUDPC (from 8 to 19 biological replicates) from two experiments are reported. Error bars show s.e.m. The asterisks indicates statistically significant differences respect to the control line EVB, according to Tukey or Games–Howell *post hoc* test (*P*=0.05). The representative leaves reproduced here were collected 30 days after inoculation.

Two independent experiments of PM inoculation were carried out on the RNAi lines TLB1 to 7, and the transgenic control EVB. Three *mlo* lines, TLB4, 5 and 6, showed a reduction of *E. necator* infection >60% at 30 d.p.i. (Figure 1; Table 1). The disease reduction of TLB6 decreased with the progression of the infection (Table 1). TLB1, 2, 3 and 7 had a level of susceptibility to PM comparable to EVB (Figure 1; Supplementary Figure S2). The leaf phenotypes in Figure 1 visualize the differences between the different lines. All the *mlo* lines showed fewer conidia at 30 d.p.i. compared with EVB and the decrease was statistically significant for TLB4, TLB5 and TLB6 (Supplementary Figure S3). Compared with EVB plants, TLB4, 5 and 6 had a reduction of 93%, 95% and 72%, respectively. Conidia counts and disease severity were, as expected, correlated (*R*=0.58; *P*⩽0.01). The reduction of conidia in TLB 4, 5 and 6 (93, 95 and 72%) was higher than the reduction of PM symptoms (68.4, 76.6 and 65.1%), indicating that the leaf diseased area had a lower concentration of conidia in TLB 4, 5 and 6 compared with EVB.

**Figure 2:**
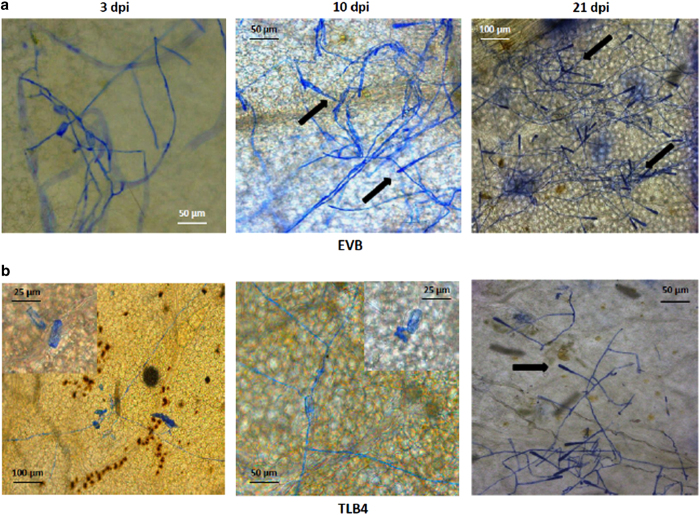
Germination of *E. necator* conidia in the control line EVB (**a**) and in the resistant transgenic line TLB4 (**b**). Microscopy images of infected leaves were taken at 3, 10 and 21 days post inoculation (d.p.i.) with powdery mildew. Insert at high magnification highlights the germination of an *E. necator* conidia at 3 and 10 d.p.i. The arrows indicate the conidiophores.

**Figure 3:**
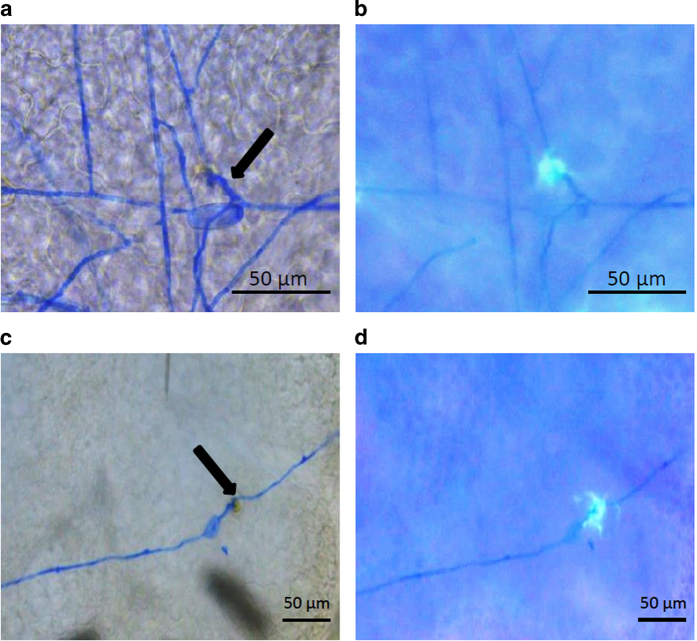
Microscopic visualization of powdery mildew infection in the control line EVB (**a**, **b**) and in the resistant transgenic line TLB4 (**c**, **d**). Microscopy images were taken with bright-field (**a**, **c**) and fluorescence (**b**, **d**) microscope at 3 days post inoculation (d.p.i.). One representative picture is reported for each line and arrows indicate germinated conidia. Scale bars, 50 μm.

Line TLB4 was characterized by histological analysis, demonstrating a reduced progression of PM infection compared with EVB (Figure 2). In EVB, conidiophores appeared at 10 and 21 d.p.i. and they were present all over the leaf surface (Figure 2a), whereas on TLB4 leaves they were visible in a limited number only at 21 d.p.i. (Figure 2b). Histological analysis revealed the accumulation of autofluorogenic phenolic compounds possibly associated to host cell response and papillae formation in both TLB4 and EVB plants at 3 d.p.i. (Figure 3).

An experiment was designed to test the cross-reaction of *mlo* lines to fungal pathogens different from PM. Three *mlo* lines (TLB1, 3 and 4) and the EVB control were inoculated with the downy mildew causal agent *Plasmopara viticola*. None of the plants were resistant and all plants showed statistically comparable levels of susceptibility to the pathogen (Supplementary Figure S4).

### Expression of *MLO* genes in the *mlo* transgenic lines

**Figure 4:**
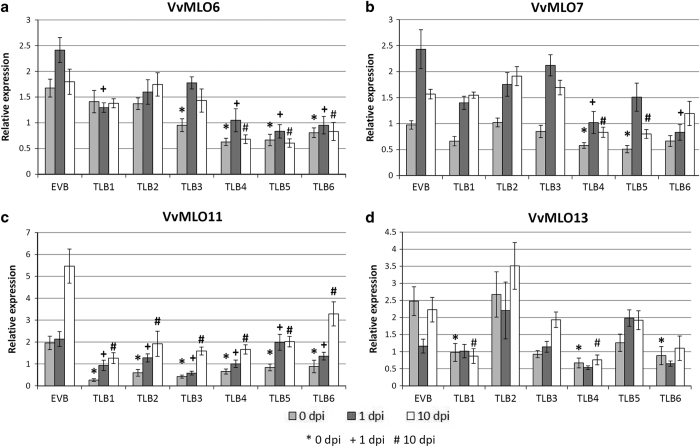
Gene expression of four grapevine *MLO* genes in the *mlo* lines TLB1, 2, 3, 4, 5 and 6, and in the control line EVB, following inoculation with *E. necator*. Expression of *VvMLO6* (**a**), *VvMLO7* (**b**), *VvMLO11* (**c**) and *VvMLO13* (**d**) was analyzed before (0 d.p.i.; light gray), 1 (dark gray) and 10 (white) days post inoculation. The mean scores of five to nine plants pooled from the two experiments are reported for each line. Error bars show s.e.m. For each time point, symbols highlight significant differences respect to the control EVB, according to Tukey or Games–Howell *post hoc* test (*P*=0.05): *for 0 d.p.i.,+ for 1 d.p.i. and # for 10 d.p.i.

The lines TLB1 to 6 and the EVB control were considered in a gene expression analysis. The results concerned four genes member of clade V and supported the off-target cross-silencing, as well as, some variability among samples of different time points (Figure 4). Lines TLB1, 2 and 3, all resulting from transformation (iii) (KD-*VvMLO11*), as expected had the target gene *VvMLO11* silenced. TLB1 showed also knockdown of *VvMLO13* and TLB3 of *VvMLO6* (Table 2). Lines TLB4, 5 and 6 derived from transformation (iv) (KD-*VvMLO13*) showed more off-target silencing: in TLB4 and 6, all four clade V *MLO* genes were, to some degree, significantly knocked down, whereas in TLB5 the expression of genes *VvMLO6*, *7* and *11* was reduced (Table 2).

### Gene expression analysis of the *mlo* line TLB4

**Figure 5:**
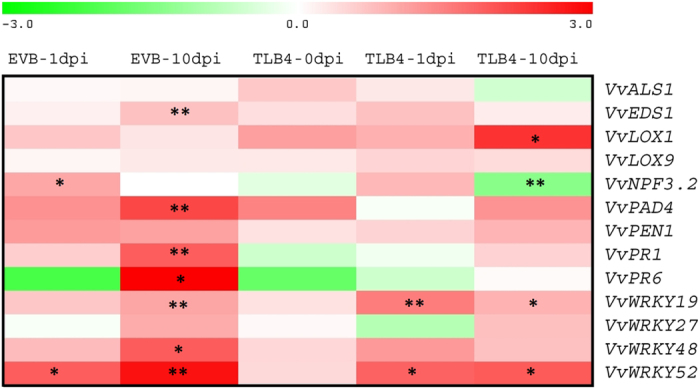
Relative expression of 13 grapevine genes at three time points in the control line EVB and in the resistant line TLB4. The color scale indicates the expression values relative to the control EVB at 0 d.p.i, used as reference for data normalization. The asterisks highlight statistically significant differences according to Fisher *post hoc* test. One and two asterisks indicate significance at *P*=0.05 and *P*=0.01, respectively. The image was prepared with the Multiexperiment Viewer software with the Log2 of relative expression data.

The expression profile at three time points of 13 genes not belonging to the *MLO* gene family and modulated by PM infection was carried out for the resistant line TLB4 and compared with the EVB line (Figure 5; Supplementary Table S4). The reason to choose TLB4 over the other resistant lines was that, in this line, all four *MLO* clade V genes were knocked down and the knockdown was more intense than TLB5 and 6. In EVB, seven of the genes tested were upregulated at 10 d.p.i. In TLB4, fewer genes were upregulated and the increase of expression was limited in terms of fold change (Figure 5; Supplementary Table S4). Moreover, three genes were downregulated in TLB4 after inoculation, namely, *VvPR6* (*pathogenesis related*) at 1 d.p.i. and *VvNPF3.2* (*nitrate transporter/peptide transporter family*) and *VvALS1* (*acetolactate synthase*) at 10 d.p.i. It is noteworthy that, before the inoculation, there were no differences in expression between TLB4 and the control EVB (Figure 5; Supplementary Table S4).

## Discussion

Loss-of-function mutations of *MLO* genes reduce susceptibility to PM in barley,^63^ *A. thaliana*,^21^ pea,^18^ tomato,^22^ wheat^23^ and pepper.^24^ Because in dicots not all Clade V MLO S-genes are implicated in PM susceptibility,^21,22,29,30^ the aim of this work was to identify which of the clade V *MLO* genes of grapevine have a role in PM susceptibility and can be inactivated to develop resistant genotypes. Out of 26 transgenic lines tested, six from gene transfers (iii) (KD-*VvMLO11*) and (iv) (KD-*VvMLO13*) supported significant gene knockdown. In the regenerated lines obtained from gene transfers (i) (KD-*VvMLO6*) and (ii) (KD-*VvMLO7*), reduction of expression was not evident. It cannot be excluded that this was due to the short RNAi fragments present in the constructs.^64^ The detection of off-target silencing in five of the six mentioned lines was expected, as clade V *MLO* genes have high levels of sequence identity (36–60%, 46% on average^29,30^). To find a balance between specificity (short RNAi fragments) and effectiveness (long RNAi fragments) is particularly difficult in gene families with high sequence similarity.^65^ As the aim was to study the effect of the knockdown of four *MLO* genes quite similar to each other, we opted for long RNAi fragments, so that off-target silencing was not only expected but also desired.

Knockout and knockdown of *MLO* genes may induce pleiotropic phenotypes, such as necrotic spots on leaves and reduced grain yield in barley,^20^ slow growth in *A. thaliana*^21^ and reduced plant size in pepper.^24^ In grapevine, no pleiotropic phenotypes were observed under the experimental conditions adopted.

Lines TLB4, 5 and 6, which showed clear resistance to PM, allowed the investigation of the link between resistance and the expression of specific *MLO* genes. *VvMLO11* expression was significantly reduced in susceptible and resistant *mlo* lines: it is concluded that its knockdown was not directly linked to grapevine susceptibility to PM. *VvMLO6* was significantly silenced in the resistant lines TLB4, 5 and 6, and in the susceptible line TLB3. As for *VvMLO11*, the knockdown of *VvMLO6* in both susceptible and resistant lines indicates that this should not be an S-gene. Similarly to *VvMLO6*, *VvMLO13* was knocked down in the resistant lines TLB4 and 6, but also in the susceptible line TLB1. *VvMLO7* was knocked down only in the three resistant lines TLB4, 5 and 6, but it was always knocked down together with other two or three *MLO* genes, as there was no line showing the knockdown of *VvMLO7* only. Therefore, the knockdown of multiple *MLO* genes provided resistance to PM. *VvMLO6* was also knocked down in all the resistant lines with a reduction of expression of 58–65%, whereas the reduction of its expression was of only 29% in the susceptible line TLB3, indicating that it can contribute to PM resistance. There are no information available about how the reduction of the expression of an S-gene affects disease severity: it could be a linear relationships, meaning that the reduction of expression causes a proportional reduction of disease severity, or there could be a threshold above which the knockdown, even if statistically significant, does not cause any reduction of disease severity. Given the weak knockdown of *VvMLO6* in TLB3 (29%), it is possible that this hypothetical threshold was not surpassed, therefore we cannot rule out the possibility of a role for *VvMLO6*. This would be particularly surprising, as there are no precedence of *MLO* genes acting as S-genes without being upregulated upon PM inoculation, such as *VvMLO6*.^29,30^ In conclusion, *VvMLO6* and *VvMLO7* are the main candidates for causing PM susceptibility in *V. vinifera,* with a possible additive activity. A similar scenario was observed in *A. thaliana*, where the simultaneous knockout of three *MLO* genes is necessary to obtain complete PM resistance: knockout of *AtMLO2* results in a moderate level of resistance, whereas the knockout of *AtMLO*6 and *AtMLO1*2, alone or combined, does not decrease the intensity of the infection.^21^ When *AtMLO2* is knocked out together with *AtMLO6* or *AtMLO12*, the level of resistance rises to become complete when the three genes are knocked out together.^21^ In grapevine, *VvMLO7* is the best candidates to act as *A. thaliana AtMLO2*, whereas *VvMLO6* and *VvMLO11* might have an additive synergistic role in PM susceptibility, as their expression was also significantly reduced in all three resistant lines. In *A. thaliana,* the knockout of three *MLO* genes induces complete resistance,^21^ a situation not observed in grapevine, in agreement with the incomplete silencing of *MLO* genes obtained by the RNAi approach. A complementation test carried out in *A. thaliana mlo* triple mutant showed that *VvMLO11* and *VvMLO13* induce susceptibility to PM, whereas *VvMLO7* has only a partial effect and *VvMLO6* has no effect at all.^66^ However, single and double *VvMLO11* and *VvMLO13* knockdown mutants of *V. vinifera* obtained by RNAi did not show significant reduction of PM penetration.^15^ Accordingly, our data indicated only a putative additive effect provided by *VvMLO11* and no role at all for *VvMLO13.* The differences observed with the results carried out by Feechan *et al.*^66^ can be explained by the fact that they operated in a heterologous system (*A. thaliana)* not reproducing with fidelity the PM infection of grapevine plants.

The precise mechanism through which the reduction of *MLO* genes expression results in resistance to PM pathogens is not completely clear. Resistance seems linked to secretory vesicles traffic^2,35^ and to the formation of cell wall appositions called papillae.^21^ These structures consists of a callose matrix enriched in proteins and autofluorogenic phenolics compounds,^54^ and their formation depends on endomembrane transport.^67^ Our results showed the accumulation of autofluorogenic compounds at the infection sites of transgenic and control lines that reflects the host response and the papilla formation. This first observation suggested that papillae were accumulated in both TLB4 and EVB lines. A difference in dimension was noted between the two lines, but further investigations are required to clear whether this is a direct consequence of *MLO* knockdown or a random event. Particularly, the use of specific staining protocols^68^ would allow to better characterize the composition, dimensions and timing of papilla accumulation in resistant and susceptible lines. It is known that the defense response based on papillae differs between resistant and susceptible genotypes in timing of formation, composition and size.^67–69^ Particularly, Chowdhury *et al.*^68^ showed that the difference between effective and non-effective papillae is due to the higher concentration of callose, cellulose and arabinoxylan of the effective ones. The MLO protein has been proposed to be a negative regulator of vesicle-associated and actin-dependent defense pathways at the site of attempted PM penetration.^27^ Furthermore, Miklis *et al.*^35^ proposed that, once MLO proteins are under the control of the fungus, actin filaments serve the purpose of supplying nutrients for the growing hyphae through vesicular transport. The pathogen may be able to control the transport of material to the cell wall, with the purpose of changing the composition of the papillae and turning them from effective to non-effective.

The formation of papillae is not the only process instigated by the activity of *MLO* genes. To understand the effect of *MLO* knockdown on other genes involved in plant–pathogen interaction, the expression of 13 genes known to be differentially expressed after PM inoculation^55–57,66,70–74^ was analyzed. The knockdown of *MLO* genes in the TLB4 line did not affect the expression levels of the 13 considered genes as compared with the EVB line in absence of PM infection. At 1 d.p.i, the response of the considered genes was very limited in both the lines tested, suggesting that the infection was not sufficient to trigger the response. Conversely, some differences between TLB4 and EVB lines were observed at 10 d.p.i. As a general trend, seven genes (*VvEDS1*, *VvPAD4*, *VvPR1*, *VvPR6*, *VvWRKY19*, *VvWRKY48* and *VvWRKY52*) were upregulated in EVB, reflecting the reaction of grapevine to PM. Conversely, only four genes (*VvLOX1*, *VvNPF3.2*, *VvWRKY19* and *VvWRKY52*) were activated in TLB4, suggesting that the less severe PM infection was not sufficient to activate the defense pathways with the same intensity of the susceptible control EVB. Particularly, *VvNPF3.2*, a nitrite/nitrate transporter, is known to be upregulated in grapevine infected with *E. necator*^70^ and it was downregulated in TLB4 at 10 d.p.i, indicating that only a severe infection could elicit *VvNPF3.2* upregulation in this line. Three genes were upregulated at 10 d.p.i. in the control EVB but not in TLB4, namely, *VvPR1*, *VvPR6* and *VvEDS1* (*enhanced disease susceptibility*). The two pathogenesis-related genes are involved in plant defense and are known to be upregulated in PM-infected grapevine leaves pretreated with a salicylic acid (SA) analog,^57^ whereas *VvEDS1* is a grapevine defense gene involved in the SA pathway,^56^ a plant growth regulator that activates pathogenesis-related genes and induces disease resistance.^71^ This may indicate that only a severe *E. necator* infection triggers the plant defense depending on SA. However, the expression profiles observed in this two lines need to be confirmed by further gene expression studies that comprise more transgenic control and knockdown lines.

The knockout of *MLO* genes increased susceptibility to other pathogens in barley^75,76^ and *A. thaliana*.^21^ Not surprisingly, the infection with *P. viticola*, an obligate biotroph fungus such as *E. necator*, revealed that the knockdown of *MLO* genes did not change the susceptibility of grapevine to downy mildew, supporting the conclusion that *MLOs* S-genes are specific for *E. necator* and are not involved in the plant interaction with *P. viticola*.

This work provides crucial information that can be used in breeding grapevine varieties resistant to *E. necator*. The tagging via genome editing of the *MLO* genes identified in this paper should result in knockout mutants highly resistant to PM. Alternatively, the search in *V. vinifera* and in wild species of non-functional *MLO* alleles should contribute to the creation of durable resistance.

## Figures and Tables

**Table 1:** Disease reduction of seven RNAi lines transformed with *MLO* knockdown constructs.

	*Gene transfer*	*Number of plants*	*Disease reduction (%)* a			*Average reduction (%)*
			*14 d.p.i.*	*22 d.p.i.*	*30 d.p.i.*	
TLB1	iii	8	22.8	32.3	34.3	29.8
TLB2	iii	15	49.2	37.2	23.8	36.8
TLB3	iii	15	17.9	14.8	2.0	11.6
TLB4	iv	19	60.8	71.7	72.8	68.4
TLB5	iv	14	76.7	79.1	74	76.6
TLB6	iv	11	71.8	63.1	60.3	65.1
TLB7	iii	13	−8.0^#^	−21.5^b^	−21.2^b^	−16.9^b^

Abbreviations: EVB, empty vector Brachetto; RNAI, RNA interference.

a Line EVB was the control (12 replicates). Disease reduction was calculated as disease severity of EVB−disease severity of the transgenic line divided by disease severity of EVB×100.

b The negative values of TLB7 indicate higher level of infection compared with EVB.

**Table 2:** Relative level of expression (RE%)^a^ of *VvMLO6*, *7*, *11* and *13* in transgenic lines TBL1 to 7

	*VvMLO6*	*VvMLO7*	*VvMLO11*	*VvMLO13*
TLB1	67	72	25**	49**
TLB2	79	94	40**	156
TLB3	71*	93	27**	69
TLB4	38**	49**	34**	33**
TLB5	35**	55**	50**	88
TLB6	42**	53**	55**	45**
TLB7	83	85	51	57

*, **indicates statistically significant difference at *P*=0.05 and *P*=0.01, respectively, according to the Tukey *post hoc* test.

a Each RE% value is the average of time points 0, 1 and 10 d.p.i. and of two experiments. RE%=(RE of *mlo* line/RE of control EVB)×100.
