# Supplementary figures and images for: Knockdown of MLO genes reduces susceptibility to powdery mildew in grapevine
Source: Hortic Res. 2016 Apr 20;3:16016–. doi: 10.1038/hortres.2016.16 (PMC4935963; doi:10.1038/hortres.2016.16)

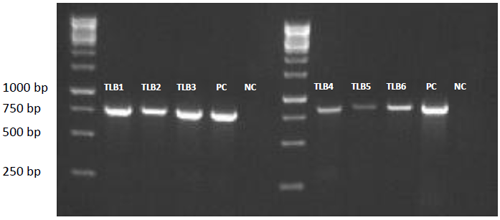

Supplement: Supplementary Figure 1 [file hortres201616-s1.tiff]

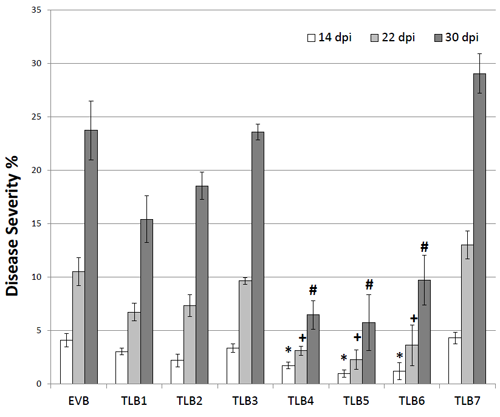

Supplement: Supplementary Figure 2 [file hortres201616-s2.tiff]

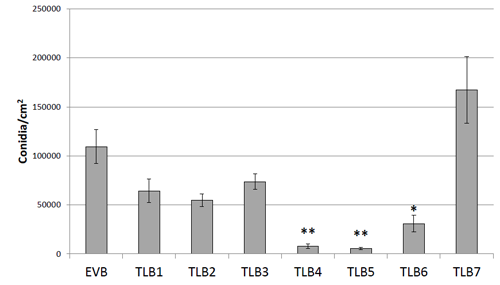

Supplement: Supplementary Figure 3 [file hortres201616-s3.tiff]

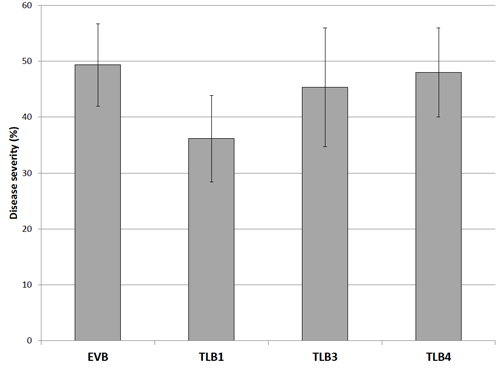

Supplement: Supplementary Figure 4 [file hortres201616-s4.tiff]
