# Supplementary material for: Knockdown of MLO genes reduces susceptibility to powdery mildew in grapevine
Source: Hortic Res. 2016 Apr 20;3:16016–. doi: 10.1038/hortres.2016.16 (PMC4935963; doi:10.1038/hortres.2016.16)
Supplement: Supplementary Information [file hortres201616-s5.doc]

**SUPPORTING INFORMATIONS**

**Table S3.** Summary of gene transfers results

| **Gene transfer** | **Knock-down construct** | **Regenerant lines** | **Confirmed transgenic** | **Selected** |
| --- | --- | --- | --- | --- |
| i | *VvMLO6* | 1 | 1 | / |
| ii | *VvMLO7* | 3 | 1 | / |
| iii | *VvMLO11* | 13 | 10 | TLB1, TLB2, TLB3, TLB7 |
| iv | *VvMLO13* | 13 | 11 | TLB4, TLB5, TLB6 |
| Empty Vector | / | 7 | 6 | EVB |

**Table S4. Relative expression of 13 grapevine genes at three time points in EVB and in TLB4**

|  | **0 dpi EVB** | **1 dpi EVB** | **10 dpi EVB** | **0 dpi TLB4** | **1 dpi TLB4** | **10 dpi TLB4** |
| --- | --- | --- | --- | --- | --- | --- |
| ***VvALS1*** | 1.00 | 1.06 | 1.09 | 1.58 | 1.22 | 0.69 |
| ***VvEDS1*** | 1.00 | 1.13 | 1.67 | 1.32 | 1.66 | 1.19 |
| ***VvLOX1*** | 1.06 | 1.60 | 1.25 | 2.20 | 1.93 | 5.40 |
| ***VvLOX9*** | 1.12 | 1.09 | 1.20 | 1.22 | 1.42 | 1.34 |
| ***VvNPF3.2*** | 1.02 | 2.06 | 0.99 | 0.79 | 1.79 | 0.38 |
| ***VvPAD4*** | 1.29 | 2.46 | 4.51 | 2.78 | 0.94 | 2.42 |
| ***VvPEN1*** | 1.13 | 2.29 | 2.16 | 1.28 | 1.43 | 1.84 |
| ***VvPR1*** | 1.06 | 1.51 | 3.75 | 0.67 | 0.89 | 1.45 |
| ***VvPR6*** | 1.07 | 0.22 | 12.08 | 0.29 | 0.66 | 1.04 |
| ***VvWRKY19*** | 1.02 | 1.58 | 2.07 | 1.27 | 2.91 | 1.88 |
| ***VvWRKY27*** | 1.27 | 0.94 | 1.99 | 1.06 | 0.55 | 1.69 |
| ***VvWRKY48*** | 1.21 | 1.74 | 3.78 | 1.38 | 2.31 | 1.67 |
| ***VvWRKY52*** | 1.21 | 3.76 | 7.00 | 1.37 | 3.61 | 3.84 |

**Figure S1.** Presence of the construct in the transgenic lines selected for powdery mildew inoculation. The positive control (PC) was a colony PCR on the *Agrobacterium tumefaciens* strain used for gene transfer. The negative control (NC) was the DNA of wild-type ‘’Long-Cluster Brachetto’’. The expected fragments length were 714 bp for lines TLB1 to 3 (210 for the 35s promoter and 504 bp for the insert), and 837 bp for lines TLB4 to 6 (210 for the 35s promoter and 627 bp for the insert).

**Figure S2.** Disease severity at three time point of grapevine transgenic lines inoculated with *Erysiphe necator*. The mean scores of powdery mildew severity were calculated on 8-19 biological replicates from two experiments. Error bars show standard error of the mean. For each time point, symbols highlight significant differences respect to the control EVB, according to Tukey or Games-Howell post-hoc test (P = 0.05): * for 0 dpi, + for 1 dpi and # for 10 dpi.

**Figure S3.** Number of conidia per leaf surface (cm^2^) of grapevines inoculated with *Erysiphe necator* at 30 dpi. Control (EVB) and transgenic lines (TLB1, TLB2, TLB3, TLB4, TLB5, TLB6 and TLB7). The mean values of conidia counts of 8-19 biological replicates from two independent experiments are reported. Error bars show standard error of the mean. One (P = 0.05) and two (P = 0.01) asterisks highlight statistically significant differences compared to line EVB, according to Kruskall-Wallis test.

**Figure S4.** Disease severity a 7 dpi of grapevine transgenic lines inoculated with *Plasmopora viticola*. The mean scores of downy mildew severity were calculated on 6-9 biological replicates from two experiments. Error bars show standard error of the mean. Tukey post-hoc test (P = 0.05) revealed non-significant differences among the grapevine lines.
